# Endochondral Ossification Induced by Cell Transplantation of Endothelial Cells and Bone Marrow Stromal Cells with Copolymer Scaffold Using a Rat Calvarial Defect Model

**DOI:** 10.3390/polym13091521

**Published:** 2021-05-09

**Authors:** Zhe Xing, Xiaofeng Jiang, Qingzong Si, Anna Finne-Wistrand, Bin Liu, Ying Xue, Kamal Mustafa

**Affiliations:** 1 School of Stomatology, Lanzhou University, Lanzhou 730000, China; xingz@lzu.edu.cn (Z.X.); jiangxf19@lzu.edu.cn (X.J.); siqz@lzu.edu.cn (Q.S.); 2 Department of Clinical Dentistry, Faculty of Medicine, University of Bergen, 5009 Bergen, Norway; kamal.mustafa@uib.no; 3 Department of Fibre and Polymer Technology, KTH Royal Institute of Technology, Teknikringen 56-58, SE 100-44 Stockholm, Sweden; annaf@kth.se

**Keywords:** bone marrow stromal cells, endothelial cells, copolymer scaffold, endochondral ossification, host response

## Abstract

It has been recently reported that, in a rat calvarial defect model, adding endothelial cells (ECs) to a culture of bone marrow stromal cells (BMSCs) significantly enhanced bone formation. The aim of this study is to further investigate the ossification process of newly formed osteoid and host response to the poly(L-lactide-co-1,5-dioxepan-2-one) [poly(LLA-co-DXO)] scaffolds based on previous research. Several different histological methods and a PCR Array were applied to evaluate newly formed osteoid after 8 weeks after implantation. Histological results showed osteoid formed in rat calvarial defects and endochondral ossification-related genes, such as dentin matrix acidic phosphoprotein 1 (Dmp1) and collagen type II, and alpha 1 (Col2a1) exhibited greater expression in the CO (implantation with BMSC/EC/Scaffold constructs) than the BMSC group (implantation with BMSC/Scaffold constructs) as demonstrated by PCR Array. It was important to notice that cartilage-like tissue formed in the pores of the copolymer scaffolds. In addition, multinucleated giant cells (MNGCs) were observed surrounding the scaffold fragments. It was concluded that the mechanism of ossification might be an endochondral ossification process when the copolymer scaffolds loaded with co-cultured ECs/BMSCs were implanted into rat calvarial defects. MNGCs were induced by the poly(LLA-co-DXO) scaffolds after implantation, and more specific in vivo studies are needed to gain a better understanding of host response to copolymer scaffolds.

## 1. Introduction

Severe musculoskeletal injury, which are caused by trauma, degeneration or skeletal abnormalities, may result in large bone defects [1]. Bone repair remains a major challenge, therefore, reconstructive therapies are needed to assist the healing process. Autologous bone graft is the main approach for bone repair and regeneration, due to its greater osteogenic capacity and inherent biocompatibility [2,3]. However, the limitation of supply and surgical morbidity at the donor site such as infections and paresthesia are related to limited use of autologous bone graft [4,5].

Bone tissue engineering (BTE) has been proposed as an alternative method for bone repair to overcome the drawbacks of autogenous bone graft. BTE consists of three elements: scaffolds, cells, and growth factors [6]. Mesenchymal stem cells (MSCs), which have been widely used in tissue engineering, can differentiate into multiple lineages such as osteoblasts, chondrocytes, and myocytes [7]. In a meta-analysis study, combined cell transplantation of MSCs and endothelial progenitor cells, compared with cell transplantation of MSCs alone, can significantly stimulate angiogenesis and bone regeneration [8]. Materials play a crucial role in BTE. They not only provide support for cells’ attachment and proliferation, but also possess biocompatibility with good osteoconductivity and osteoinductivity [9,10]. Cells can be seeded into biological scaffolds to establish tissue-engineered constructs for tissue repair.

Bone is a complex hard tissue that can provide support for the body and protect organs. Bone tissue can be categorized into compact bone and cancellous bone. Bone formation relies on two essential processes: intramembranous ossification and endochondral ossification. Intramembranous ossification directly converts mesenchyme to bone, whereas endochondral ossification is a process that mesenchyme transforms into cartilage eventually replaced by bone [11]. Attempts on bone formation have predominantly focused on intramembranous ossification in BTE. Intramembranous ossification can be achieved in intra-oral osseous defects of patients through implantation of bone substitute formed by combination of bone marrow stem cells (BMSCs) and materials; however, it has not reached its full potential due to lack of sufficient vascularization of the implants [12]. Blood vessel is essential for cells in order to obtain oxygen and nutritional supply [13,14]. Endochondral ossification is beneficial for vascularization at the implant site as hypertrophic chondrocytes can secrete angiogenic factors [15]. Therefore, endochondral ossification is a promising mechanism in BTE.

In our previous study, a cross talk between endothelial cells (ECs) and BMSCs played a crucial role in bone regeneration. Our previous finding has shown that significant upregulation of alkaline phosphatase was observed when adding less than 20% of ECs to BMSCs in vitro [16]. In an in vivo study, the results clearly illustrated that co-cultured ECs/BMSCs in the poly(L-lactide-co-1,5-dioxepan-2-one) [poly(LLA-co-DXO)] scaffolds could enhance the osteogenic potential of BMSCs [17]. Nevertheless, the mechanism of ossification triggered by co-cultured cells still lacks conclusive evidence.

Rapid development has been achieved in the field of materials in the past, thus, many better biocompatible materials have been discovered and applied for BTE. Clinical application depends on biocompatibility and biodegradation of materials [11]. It has been reported that degradable poly(LLA-co-DXO) scaffolds were good for cell attachment, proliferation, and osteogenic differentiation in vitro in previous studies [18,19]. In addition, degradation of the poly(LLA-co-DXO) scaffolds was tested in vitro. When poly(LLA-co-DXO) was placed in phosphate-buffered saline (PBS) with the presence of proteinase K, poly(LLA-co-DXO) was gradually degraded and showed a significant mass loss. Meanwhile, acidic degradation products such as lactic acid were produced, resulting in a decrease in the pH of the incubation solution [20]. Acidic degradation products of synthetic polymers affected the pH of surrounding environment and caused inflammatory response in surrounding tissue. For example, lactic acid as the end product of poly(lactide-co-glycolide) exerted an influence on immune response [21]. In previous studies, host response to the poly(LLA-co-DXO) scaffolds has not been reported in detail in in vivo experiments.

Host response reflects the integration between materials and host cells; however, excessive host response may prolong tissue healing or lead to the failure of tissue regeneration. Macrophages represented a major cellular component of host response and took charge of destruction and integration of materials [22]. It is generally believed that macrophages are the precursor cells of multinucleated giant cells (MNGCs) [23]. Macrophages can fuse into MNGCs during chronic inflammation owing to failing in phagocytizing foreign particles [24]. Macrophages and MNGCs, observed at material–tissue interfaces, are dominant host cells responding to material implantation [25]. It has been reported that there were large number of MNGCs surrounding failed implants [26,27].

Therefore, the aim of this in vivo study is to further investigate ossification process triggered by the BMSC/EC constructs through histological analysis and at the molecular level. Host response to the poly(LLA-co-DXO) scaffolds was also observed and presented.

## 2. Materials and Methods

### 2.1. Preparation of Scaffolds

The scaffolds were fabricated at the Department of Fibre and Polymer Technology, KTH Royal Institute of Technology, Sweden. The method of fabrication has been well described in our previous studies [17,28], briefly, L-lactide (LLA) and 1,5-Dioxepan-2-one (DXO) were prepared and used as monomers for synthesizing the copolymer poly(LLA-co-DXO). Stannous 2-ethylhexanoate (SnOct2, Sigma Aldrich, St. Louis, MO, USA) was used as catalysts and ethylene glycol as initiator. The bulk polymerization was proceeded at 110 °C for 72 h. The resulting polymer was precipitated three times using cold hexane and methanol.

Poly(LLA-co-DXO) scaffolds were fabricated by a solvent-casting-particulate-leaching technique [28]. Briefly, poly(LLA-co-DXO) was dissolved in chloroform to form 4% (*w/v*) polymer solution, which was poured into a glass mold containing sodium chloride. The ratio of poly(LLA-co-DXO) and sodium chloride was 10:1 in weight. The chloroform evaporated and samples were subsequently punched out from the sodium chloride–polymer composite. The punched-out samples were poured into a water bath, and the water was repeatedly changed until all sodium chloride was dissolved. Finally, the porous poly(LLA-co-DXO) scaffolds were vacuum dried and sterilized using electron beam radiation. The pore sizes of scaffolds ranged from 90 to 400 μm, and the porosity of scaffolds was about 90%.

### 2.2. Cell Culturing, Cell Seeding

The biological experiments were performed at the University of Bergen. The procedure has been described in our previous study [17]. Briefly, BMSCs were isolated from the bone marrow from femurs of two male healthy Lewis rats and incubated at 37 °C in a high humidity environment containing 5% CO_2_. Endothelial Cell Growth Medium-2 (EBM-2^®^) (Lonza, Basel, Switzerland) was applied to make part of harvested BMSCs to differentiate into ECs. ECs were characterized by a flow cytometer (Accuri^®^ C6, Tucson, AZ, USA) using CD31 and Flk-1. Before cell seeding, BMSCs and ECs were trypsinized from culture flasks. BMSCs (5 × 10^5^ cells) and BMSCs/ECs (5 × 10^5^ BMSCs and 1 × 10^5^ ECs) were seeded onto the top of the prewetted poly(LLA-co-DXO) scaffolds, respectively. Then, cells on the top were distributed into the pores of the scaffolds with the help of an orbital shaker (Eppendorf^®^, Hamburg, Germany). These cell/scaffold constructs were cultured overnight at 37 °C and 5% CO_2_ to allow the cells to attach and subsequently incubated in spinner flasks (Wheaton Science, Millville, NJ, USA) for 1 week before surgery.

### 2.3. Graft Implantation

The animal experiment was approved by the Norwegian Animal Research Authority (ID1572). After anesthesia with isofluorane (Isoba vet^®^, Schering-Plough, Eiksmarka, Norway) combined with NO_2_ and O_2_, the skin, the temporalis muscles and the periosteum were reflected to expose the operative region, and two symmetrical calvarial defects were created by trephine burr in twelve three-month-old Lewis rats. The symmetrical defects of nine rats were implanted with BMSC/EC/Scaffold constructs (CO group) and BMSC/Scaffold constructs (BMSC group), respectively. The remaining rats were used for experiments as controls. One side was implanted with scaffold without cells (Scaffold group), and the other side was not treated (empty group). After implantation with grafts, the wounds were closed with sutures (Vicryl Plus 5-0). The rats were sacrificed by euthanasia with an overdose of CO_2_ at week 8 after implantation and the calvarias with grafts were retrieved from the sacrificed rats. In this study, specimens were further processed with staining and PCR Array.

### 2.4. Histological Evaluation

The specimens, harvested from four groups, were fixed with 4% paraformaldehyde (Merck, Kenilworth, NJ, USA). After being decalcified in decalcification solution, which contained 10% EDTA for four weeks, the samples were embedded with O.C.T.^TM^ compound (Sakura, Tokyo, Japan). Cryosections were processed as described before [17]. Cryosections with a thickness around 8 μm were prepared and stained with Masson’s trichrome, safranin O-fast green, and hematoxylin and eosin (HE).

### 2.5. Immunofluorescence Staining for Collagen Type II

The deposition of collagen type II was evaluated by immunofluorescence technique. The primary antibody against collagen type II (sc-52658, Santa Cruz Biotechnology, Santa Cruz, CA, USA) diluted 1:200 were applied for 1 h at room temperature after a pretreatment with normal goat serum (sc-2043, Santa Cruz Biotechnology, Santa Cruz, CA, USA) diluted 1:50 in PBS for 30 min to block non-specific binding, followed by goat anti-mouse IgG-rhodamine (sc-2092, Santa Cruz Biotechnology, Santa Cruz, CA, USA) diluted 1:500 for 1 h at room temperature. Lastly, sections were treated with 4’, 6-diamidino-2-phenylindole (DAPI, Thermo Fisher Scientific, Waltham, MA, USA) to label nuclei.

### 2.6. Immunofluorescence Staining for Osteocalcin (OC)

Immunofluorescence assay was performed to assess osteoblastic activity. According to manufacturer’s instruction, sections were incubated with the primary antibody against OC (sc-30044, Santa Cruz Biotechnology, Santa Cruz, CA, USA) diluted 1:200 overnight at 4 °C, followed by incubation with the FITC-conjugated goat anti-rabbit IgG (H+L) secondary antibody (65-6111, Thermo Fisher Scientific, Waltham, MA, USA) diluted 1:500 in PBS for 3 h at room temperature. Finally, DAPI was utilized to label nuclei.

### 2.7. RT^2^ Profiler PCR Array

In addition, to investigate the difference between the CO group and BMSC group at a molecular level, paired samples from each group were processed for PCR Array analysis. Briefly, extraction of RNA was performed using an isolation kit (E.Z.N.A^®^). A spectrophotometer (ThermoScientific NanoDrop Technologies, Wilmington, DE, USA) was applied to examine RNA purity and quantification. cDNA synthesis was first carried out with the RT^2^ PCR Array First Strand Kit (SuperArray Bioscience Corporation, Frederick, MD, USA). Rat Osteogenesis RT^2^ Profiler PCR Array Kit was also purchased from SuperArray Bioscience Corporation. PCR Array was performed on an ABI StepOne^TM^ system (Applied Biosystems, Foster City, CA, USA) which was described before [16]. Data analysis was applied with an online software (http://www.superarray.com/pcr/arrayanalysis.php (accessed on 8 May 2021)) and the heat map was generated through online software. The data were analyzed by a 2^−ΔΔCt^ method [29]. SAM is a statistical technique for detecting significant gene changes.

### 2.8. Statistical Analysis

For determination of the area fraction of collagen type II and OC, the defect area was defined as region of interest. A common threshold was applied to include all high-density areas. The sum intensity was determined by software NIS-Elements AR (Nikon, Tokyo, Japan) and analyzed statistically. Graphs were created using GraphPad Prism (San Diego, CA, USA). *t*-test was used to evaluate the differences between the CO and BMSC group. Differences were considered significant if the *p* values were less than 0.05 for all statistical analysis. Experimental results were presented as mean ± standard deviation.

## 3. Results

### 3.1. Masson’s Trichrome Staining

**Figure 1:**
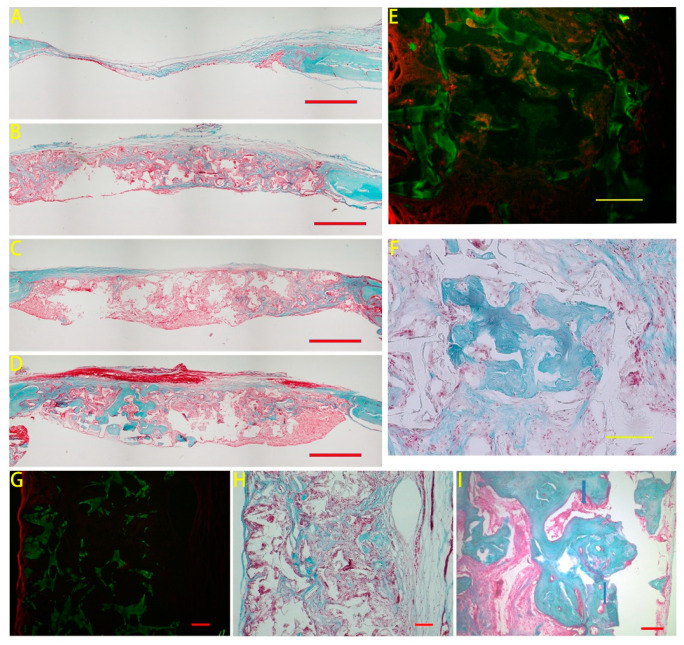
Masson’s trichrome staining results. The overview from the empty group (**A**), the Scaffold group (**B**), the BMSC group (**C**) and the CO group (**D**) was observed. Scale bar: 1mm. Newly formed osteoid (bluish green color areas) grew in the pores of the poly(LLA-co-DXO) scaffolds in the CO group (**F**,**H**). Scaffold images (green areas) were obtained by autofluorescence from the material (**E**,**G**). Blood vessels (indicated by blue arrows) were observed at the site of osteoid formation in the CO group (**I**). Scale bar: 100 μm.

The comparison between the BMSC and CO group has been illustrated in our previous study. Further histological information was illustrated in this study. The overview of the implanted area was presented in Figure 1A–D, and more osteoid could be observed from the CO group. To clearly illustrate scaffold fragments, pictures from the same areas were taken by both normal light (Figure 1F,H) and under a fluorescence filter (Figure 1E,G) by a microscope (Nikon 80i, Tokyo, Japan). According to the frame of the scaffold fragments, osteoid (in bluish green) located inside the pores of the scaffold. In a higher magnification, image from the CO group showed that blood vessels (indicated by blue arrows) were observed at the center of newly formed osteoid (Figure 1I).

### 3.2. Safranin O Staining

**Figure 2:**
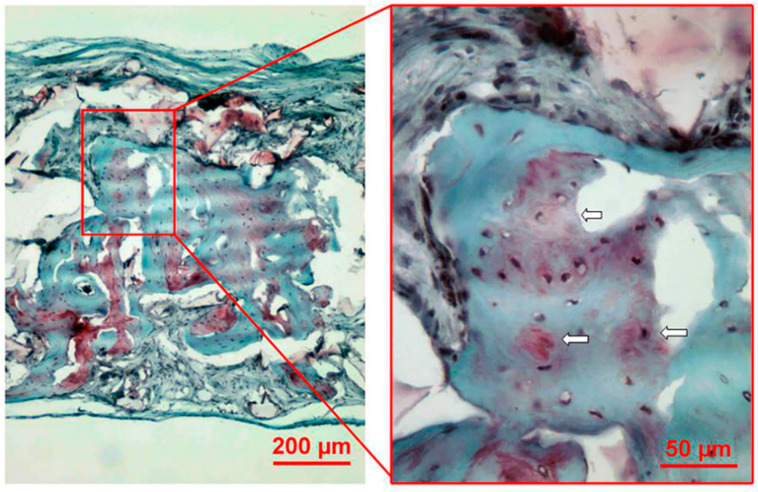
Safranin O-fast green staining results. Specimens of the CO group at week 8 after implantation were stained with a safranin O-fast green procedure. Newly formed osteoid was illustrated by the gray green color and nuclei by the gray black color. Red areas (depicted by white arrows) represented cartilage-like tissue.

Consecutive serial sections stained with the safranin O-fast green procedure were used for the histological evaluation. The results were shown in Figure 2. Newly formed osteoid illustrated by the gray green color could be observed among the debris of the copolymer scaffolds. A large number of red areas, which were positive staining for sulfated glycosaminoglycans, indicated the formation of cartilage-like tissue. In a higher magnification, cartilage-like tissue located around newly formed osteoid. Bone lacuna-like structures, in which osteocyte/chondrocyte embedded, could be seen in the newly formed osteoid and cartilage-like tissue. The results clearly illustrated an endochondral ossification process.

### 3.3. HE Staining

**Figure 3:**
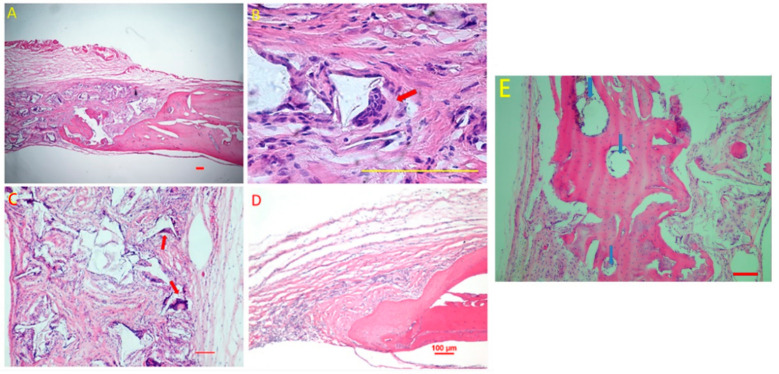
Hematoxylin and eosin (HE) staining results. New regenerated tissues, including osteoid and collagen, were observed in the region of defects (**A**,**C**,**D**). Multinucleated giant cells, indicated as red arrows (**B**,**C**), can be seen around the scaffold fragments. Sections from the empty group showed that newly formed osteoid was observed from the cutting edge, and the calvarial defects were filled with collagen (**D**). (**E**) Osteoid formed around the blood vessels in the CO group (indicated by blue arrows). Scale bar: 100 μm.

The image results in the CO group showed (Figure 3A–C) that at week 8 after implantation, new regenerated tissues, such as osteoid and collagen, were observed in the region of defects. It was predominantly soft tissues growing into the defects in the empty group (Figure 3D). In a higher magnification, MNGCs can be observed on the scaffold surface (indicated by red arrow in Figure 3B,C). Newly formed osteoid was around the blood vessels in the CO group (indicated by blue arrows in Figure 3E).

### 3.4. Expression of Collagen Type II

**Figure 4:**
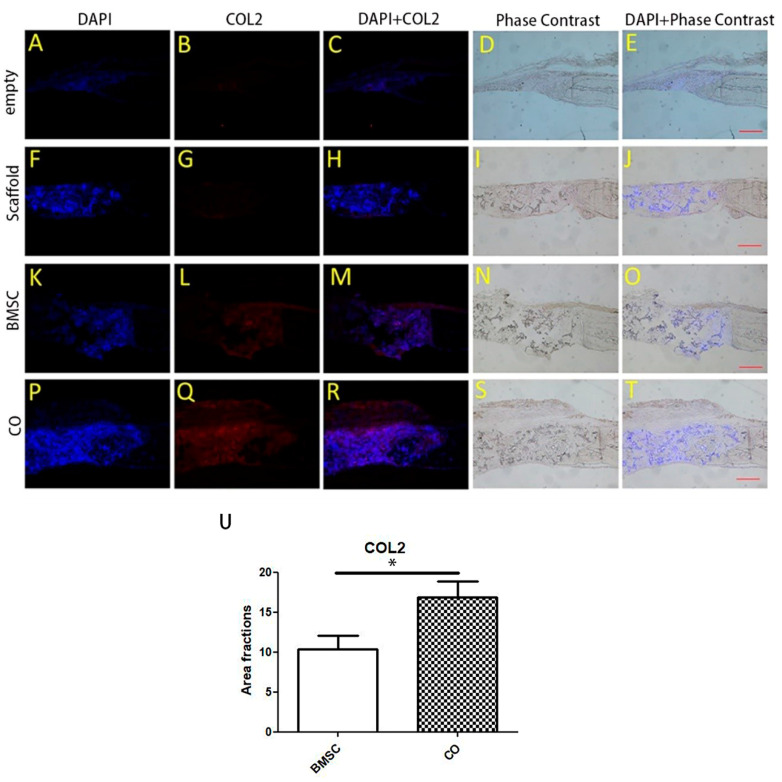
Immunofluorescence analysis for collagen type II in graft areas. Positive staining areas of collagen type II could be observed in both BMSC (**L**) and CO group (**Q**). In descending order: 1st row: images (**A**–**E**) of the empty group, 2nd row: images (**F**–**J**) of the Scaffold group, 3rd row: images (**K**–**O**) of the BMSC group, 4th row: images (**P**–**T**) of the CO group. General images (**D**,**E**,**I**,**J**,**N**,**O**,**S**,**T**) were observed by phase contrast microscope to show the overview of sections from different groups, and the remaining images were observed by fluorescence microscope to show collagen type II expression. Scale bar: 100 μm. (**U**) Area fraction of positive immunofluorescence of collagen type II in the CO and BMSC group was evaluated, and the area fraction of the CO group was more than that of the BMSC group (n = 8, * *p* < 0.05).

Immunofluorescence identified collagen type II synthesized in the CO (Figure 4Q) and BMSC group (Figure 4L). Moreover, the area fraction of collagen type II of the CO group was more than that of the BMSC group (Figure 4U).

### 3.5. OC Expression

**Figure 5:**
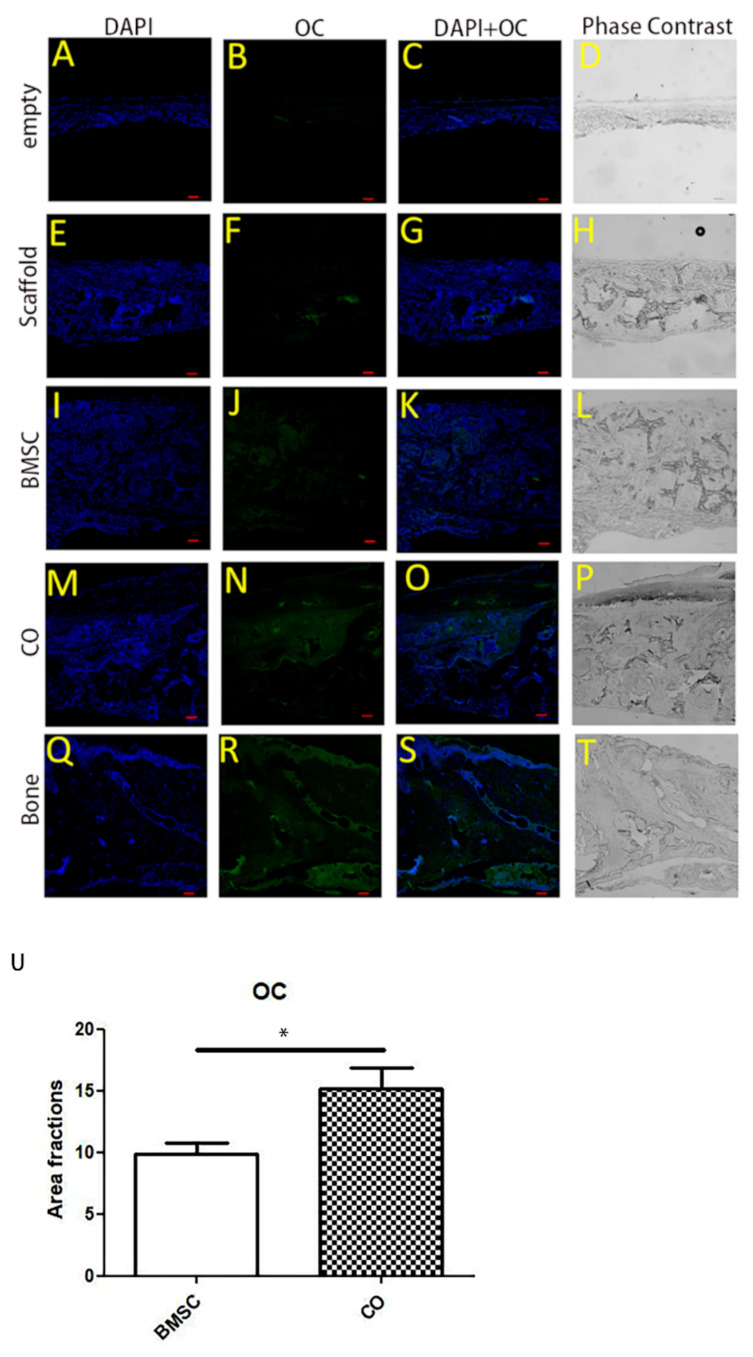
Immunofluorescence analysis for osteocalcin (OC) in graft areas. Images (**A**–**T**) showing OC expression in graft areas by immunofluorescence assay at week 8 after implantation. Immunofluorescence staining for OC was performed to assess osteoblastic activity. In descending order: 1st row: images (**A**–**D**) of the empty group, 2nd row: images (**E**–**H**) of the Scaffold group, 3rd row: images (**I**–**L**) of the BMSC group, 4th row: images (**M**–**P**) of the CO group, 5th row: images (**Q**–**T**) of the normal bone. General images (**D**,**H**,**L**,**P**,**T**) were observed by phase contrast microscope to show the overview of sections from different groups, and the remaining images were observed by fluorescence microscope to show OC expression. Scale bar: 100 μm. (**U**) Semi-quantitative analysis of OC in the CO and BMSC group. Area fraction of the images represented the protein content in the defect area. Osteoblastic activity in the CO group was stronger than that in the BMSC group (n = 9, * *p* < 0.05).

OC is used as a marker of bone formation. Sections from different groups were stained with immunofluorescence staining to show OC expression and to indicate osteoblastic activity. OC was illustrated by the green color. Osteoblastic activity in the CO group was stronger than that in the BMSC group (Figure 5U). Therefore, the results showed that more OC was expressed in the CO group, and BMSC/EC/Scaffold constructs were beneficial for bone regeneration.

### 3.6. PCR Array Analysis

**Figure 6:**
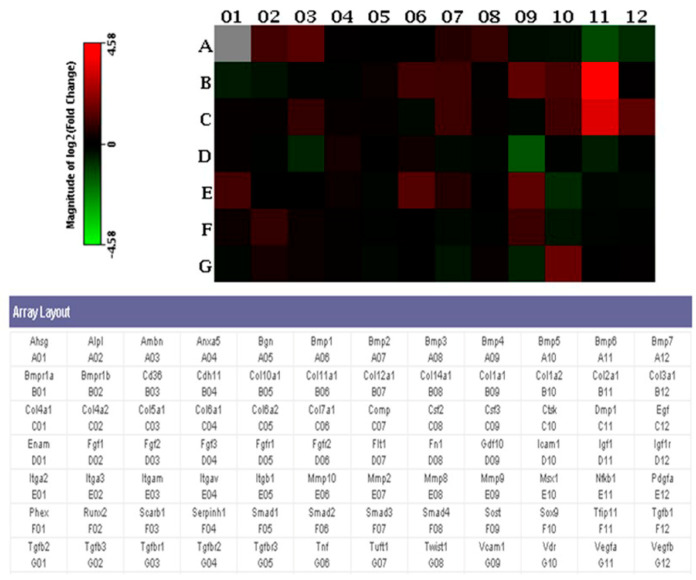
Rt^2^ Profiler Osteogenesis PCR Array. Array layout was presented, and the heat map represented the expression level of 84 genes. The red squares represented genes expressed higher in the CO group, and the green squares represented genes expressed higher in the BMSC group.

PCR Array results showed that several genes were upregulated in the CO group by adding extra ECs into the engineered constructs, compared with the BMSC group. The following findings presented in fold changes could be noticed: evaluation of osteogenic genes in the CO group showed that two genes were downregulated, and 12 genes were upregulated compared with the BMSC group (Figure 6 and Table 1).

## 4. Discussion

It has been shown that there was enhanced bone formation in the CO group [17]. In the current study, the ossification process was described by histological analysis and at the mRNA level. Moreover, host response to the poly(LLA-co-DXO) scaffolds was illustrated by the HE staining.

Our preliminary in vivo investigation of bone formation was carried out using a rat calvarial defect model. Safranin O staining was used for detection of cartilage-like tissue illustrated by the red color, and the representative image showed that cartilage-like tissue was observed in the center of the newly formed bone in the CO group. The discovery of cartilage-like tissue might prove that the repair mechanism of bone defects could be endochondral ossification. Skull was formed through intramembranous ossification during bone development [11]. The formation of the tissue-engineered osteoid was specific in the CO group whose repair mechanism differed from the nature mechanism. Calvarial defect is a simplified standard model and could not be more suitable in this case. The results from this study showed that the newly formed osteoid in the center of the scaffold was centralized with immature cartilage-like tissue and gradually formed more mature bone tissue, this is an endochondral ossification process, and this could be more meaningful in a calvarial defect model.

OC is a low-molecular-weight protein found abundantly in bone extracellular matrix [30]. The expression level of OC is closely related to bone metabolism consisting of osteogenic differentiation, bone mineralization and bone turnover [31,32,33]. Thus, OC is usually used as a marker of bone formation. OC accumulated at the sites of cartilage calcification obtained from a mineralizing chicken chondrocyte system in vitro, which suggested that OC was indeed a secretory product of hypertrophic chondrocytes near calcification sites [34]. When silencing the expression of core-binding factor alpha 1 gene led to inhibition of OC synthesis, cartilage formation was prevented at the same time [35]. In this study, gene expression of OC in the CO group may be in correlation with cartilage formation at the center of bone.

PCR Array analysis was performed in order to get a better understanding of the ossification mechanism at a molecular level. Upregulated chondrogenic-related markers were found, such as: dentin matrix acidic phosphoprotein 1 (Dmp1) and collagen type II, alpha 1 (Col2a1). In a previous study, Dmp1 has been shown to play a critical role in chondrogenesis and subsequent osteogenesis. Dmp1-deficient mice developed severe cartilage defects during postnatal chondrogenesis [36]. Immature cartilage was characterized by high level of cartilage genes, such as Col2a1 [37]. Ottavia Barbieri et al. [38] proved that negative Col2a1 transgene affected chondrocyte differentiation. Therefore, Dmp1 and Col2a1 are important regulatory genes and they are upregulated in the process of cartilage formation. In addition, several genes related to the extracellular matrix, bone remodeling or bone metabolism were activated as well, such as matrix metallopeptidase 9, matrix metallopeptidase 10, cathepsin K and vitamin D receptor. This may indicate that the newly regenerated tissue was undergoing an active remodeling from cartilage-like tissue to bone.

Histological as well as molecular data illustrated that rat calvarial defects might be regenerated through an endochondral ossification process. The importance of ECs in developing endochondral ossification was well documented [39,40]. A detailed and specific role of ECs alone during this process would be of further interest in future studies. In addition to the potential effects of ECs, other factors should be taken into consideration when analyzing this ossification process following co-culture. The different geometries of the scaffolds may control phenotypic expression in osteogenesis and chondrogenesis [41,42] and should therefore also be considered.

In this study, another aim is to illustrate host response to the poly(LLA-co-DXO) scaffolds’ implantation. Host response to the copolymer scaffolds at the implanted site can influence the clinical acceptance of scaffolds. It is well known that after a copolymer scaffold is implanted, the normal course of wound healing is altered due to cell–material surface interactions. In the current study, all the incisions of the recipient rats healed completely, and a visual examination after 8 weeks revealed no severe macroscopic tissue reactions occurred around the implanted scaffolds. Histologically, MNGCs were induced by the poly(LLA-co-DXO) scaffolds after implantation.

Ideally, the degradation of materials should be consistent with the regeneration and maturation rate of the new tissues. As the newly formed bone remodels and requires less support, the scaffolds should lose its mechanical properties and gradually degrade. Degradation products can be completely absorbed by the body [43]. In vivo, massive acidic degradation products rapidly decreased the pH value in the local environment and resorption of acidic products can lead to inflammatory response and influence tissue repair [24,44,45,46]. Degradation of the poly(LLA-co-DXO) scaffolds may contribute to host response due to buildup of acidic substance. Bone formation also linked to the pH of the extracellular microenvironment. Biologic activity of a variety of cells, including BMSCs and osteoblasts, can be influenced by acidity of culture environment [47,48]. In the CO group, BMSC/EC constructs induced more bone formation compared with other groups. It is speculated that acidic products from the copolymer scaffolds have a minimal impact on bone formation.

Based on the findings and experiment structures from our previous studies, this study mainly focused on the repair mechanism in a rat calvarial defect and results indicated that the bone defects might be repaired through an endochondral ossification process. Therefore, tissue-engineered bone formed by the poly(LLA-co-DXO) scaffold seeded with BMSCs and ECs might be suitable for repairing a large bone defect due to enhanced bone formation and its repair mechanism. However, more preclinical studies should be performed to confirm this effect from the constructs loaded with co-cultured cells in the poly(LLA-co-DXO) scaffolds. Host response is a side topic in this study. MNGCs were observed in all groups with scaffolds, but a comparison between groups was not addressed. A quantified study from the biological view should be further investigated in the future since ECs can participate in inflammatory response according to earlier publication about poly(LLA-co-DXO) scaffold [49]. Moreover, MNGCs include foreign body giant cells (FBGCs) and osteoclasts, both of which are fused by macrophage-derived mononuclear cells. FBGCs differed from osteoclasts in that osteoclasts were responsible for bone resorption in bone metabolism [50]. Consequently, more evidence is needed to distinguish FBGCs and osteoclasts, such as immunohistochemical staining to identify cell surface markers.

## 5. Conclusions

In conclusion, newly formed osteoid was evaluated via histological analysis, and endochondral ossification-related genes were upregulated in this study. Evidence indicated that the endochondral ossification process might occur in rat calvarial defects after implantation with BMSC/EC/Scaffold constructs. MNGCs may participate in host response as FBGCs in this study, and more specific in vivo studies are needed to gain a better understanding of host response to the poly(LLA-co-DXO) scaffolds.

## Figures and Tables

**Table 1:** Differentiated gene list between the CO and BMSC groups (fold changes > 2) is shown in the table.

Position	Gene	Full Name	Fold Change
A02	Alpl	Alkaline phosphatase	2.04
A03	Ambn	Ameloblastin	2.51
B09	Col1a1	Collagen type I, alpha 1	2.75
B10	Col1a2	Collagen type I, alpha 2	2.15
B11	Col2a1	Collagen type II, alpha 1	23.92
C10	Ctsk	Cathepsin K	2.03
C11	Dmp1	Dentin matrix acidic phosphoprotein 1	10.94
C12	Egf	Epidermal growth factor	2.73
E01	Itga2	Integrin alpha 2	2.04
E06	Mmp10	Matrix metallopeptidase 10	2.43
E09	Mmp9	Matrix metallopeptidase 9	2.68
G10	Vdr	Vitamin D receptor	3.08
A11	Bmp6	Bone morphogenetic protein 6	−2.50
D09	Gdf10	Growth differentiation factor 10	−2.88

## Data Availability

Not applicable.
